# A novel strategy to target metabolic dependencies in acute myeloid leukemia

**DOI:** 10.1038/s41419-025-08129-3

**Published:** 2025-11-04

**Authors:** Nithya Balasundaram, Hamenth Kumar Palani, Arvind Venkatraman, Yolanda Augustin, Shruthi Pichandi, Clement Regnault, Majeela Solomon, Abirami Rajasekaran, Mohammed Yasar, Swathy Palani Kumar, Reeshma Nair Radhakrishnan, Anu Korula, Uday Prakash Kulkarni, Eunice Sindhuvi Edison, Poonkuzhali Balasubramanian, Biju George, Aby Abraham, Sanjeev Krishna, Vikram Mathews

**Affiliations:** 1 Department of Haematology, Christian Medical College, Vellore, India; 2 Laboratory of Cellular Metabolism and Microenvironment, De Duve Institute, UC Louvain, Brussels, Belgium; 3 Clinical Academic Group in Institute for Infection & Immunity, St George’s University of London, London, United Kingdom; 4 MVLS Shared Research Facilities, University of Glasgow, Glasgow, United Kingdom; 5 St George’s University Hospitals NHS Foundation Trust, London, United Kingdom; 6 Institut für Tropenmedizin, Universitätsklinikum Tübingen, Tübingen, Germany; 7 Centre de Recherches Médicales en Lambaréné (CERMEL), Lambaréné, Gabon

**Keywords:** Cancer metabolism, Drug discovery, Cell biology

## Abstract

Acute myeloid leukemia (AML) remains difficult to cure despite recent advances. Off-target side effects of drugs currently used lead to significant morbidity and mortality. There is recognition that in AML, there is an increased dependence on OXPHOS metabolism, especially in the leukemia stem cell compartment (AML-LSC). It is also recognized that there is potential to exploit this vulnerability to treat AML. Drug re-purposing screens have suggested the potential use of artesunate (ART) to inhibit mitochondrial respiration. We have explored the potential role of ART as an additive agent in treating AML in combination with conventional therapy. Through in-vitro and in-vivo mouse model studies, we demonstrate the mechanism and efficacy of these combinations and their potential to overcome venetoclax resistance. We further demonstrate the specificity of these combinations with minimal off-target effects on normal hematopoietic stem cells (HSC). These observations warrant exploration of the additive role of ART in clinical trials.

## Introduction

Acute myeloid leukemia (AML) is the most common leukemia in adults. It has a 5-year survival rate of 25-40%, with little improvement in the past 30 years [1]. Treatment usually involves an intensive cytoreductive induction therapy combining a nucleoside analog for seven days (cytarabine) with an anthracycline for 3 days (conventional 7 + 3 regimen) or the more recently introduced low-intensity combination of venetoclax (VEN) and 5-azacytidine [2]. AML is an oligoclonal disease, genetically and morphologically heterogenous, which allows escape from single-agent targeted therapies such as FLT-3 inhibitors (Midostaurin and Gilteritinib) and IDH1/2 inhibitors (Enasidenib and Ivosidenib). This emphasizes the important role of optimizing combination therapies and treatment strategies to cure this disease.

Otto Warburg first observed that cancers share the metabolic characteristic of increased glycolysis without hypoxia [3, 4]. Although he attributed increased glycolysis to mitochondrial dysfunction, increased glycolysis is anaplerotic by replenishing substrates for synthesizing nucleosides, lipids, and proteins. In contrast, in AML, it is recognized that there is an increased dependence on mitochondrial respiration, especially in the AML leukemia stem cell compartment [5–7]. However, targeting mitochondrial dysregulation using relatively specific inhibitors of complex I (IACS-010759) and a DHODH inhibitor (beraquinar) and other less specific interventions (metformin and tigecycline) has not translated from experimental models to therapeutic benefits in patients [8–10]. Lessons learned from the clinical trial using IACS-010759, a very potent OXPHOS inhibitor, were that the resultant shift to glycolysis led to therapeutic escape (metabolic plasticity of cancers), combined with non-specific targeting, led to an unacceptable increase in blood lactate levels and lactic acidosis [11]. We have previously demonstrated and reported similar metabolic adaptability of AML cells, particularly in therapy-resistant cells [12].

These observations suggest that targeting the electron transport chain (ETC) is insufficient to lethally impact the central energy metabolism (CEM) of AML cells, particularly those resistant to conventional treatments. Defining CEM pathophysiology more comprehensively in AML may improve treatment options by suggesting rationally derived combination therapies targeting therapy-resistant cells. For example, combining FCCP with glycolytic inhibitors such as arsenic trioxide (ATO) or 2-Deoxy-D-glucose (2-DG) promoted apoptosis in otherwise ATO-resistant acute promyelocytic leukemia (APL) cells [12]. We noted a similar effect in AML cell lines and primary AML cells that were inherently resistant to ATO. The challenge with the combination of ATO + FCCP was the lack of selective specificity to target the leukemic cells [12]. As already noted in previously reported trials targeting OXPHOS metabolism, a lack of specificity for the malignancy combined with a narrow therapeutic window is likely to be ineffective and lead to unacceptable systemic toxicity [11].

We hypothesized that combining ATO (a glycolytic inhibitor) and cancer cell-specific ETC inhibitors may disrupt CEM. At the same time, the inhibition of the OXPHOS metabolic pathway alone is likely to be ineffective due to metabolic plasticity. Based on previously reported data from existing metabolism drug repurposing screens that potentially shift the energy metabolism from mitochondrial respiration to glycolysis [13], we undertook a targeted screening of molecules, preferably FDA-approved drugs, as an alternative to FCCP to selectively target the leukemic cells over their normal counterparts. CEM is regulated by the flux and availability of metabolites and cofactors, such as iron and calcium, which are often increased in cancer cells in parallel with metabolic derangements [14–17]. We also explored the impact of these additional factors on inhibiting CEM, specifically in leukemic cells.

## Results

### ART selectively disrupts the CEM of AML cells in combination with ATO

**Fig. 1:**
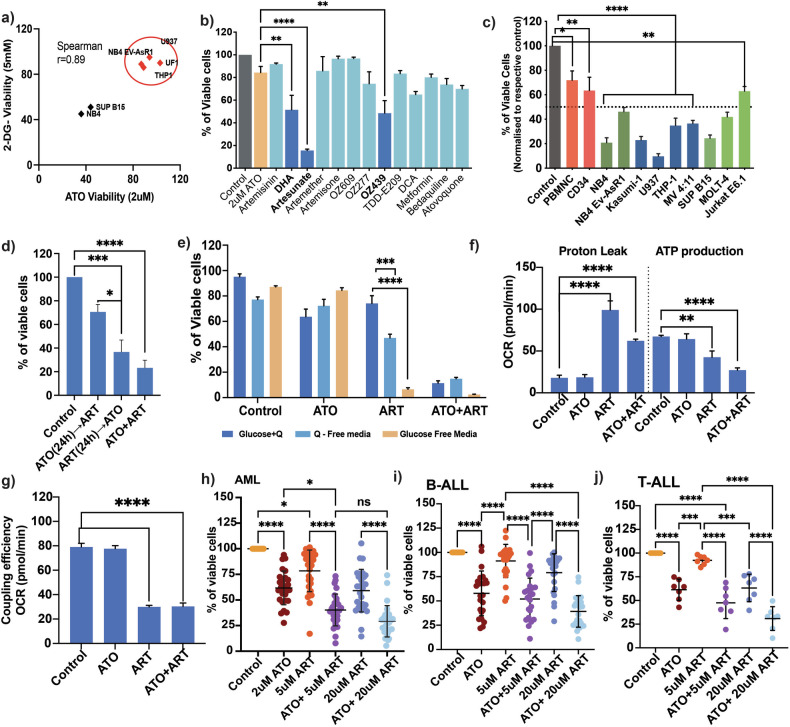
ATO and ART disrupt the CEM of AML cells with minimal off-target effects on normal cells. **a** Viability of U937 cells treated with different peroxidic antimalarials and anti-metabolites in combination with ATO (Light and dark blue represent the combination of these compounds with ATO, and a yellow bar represents single agent ATO) for 48 h (*n* = 3; ATO = 2 μM; antimalarials = 5 μM; DCA = 10 mM; Metformin = 5 mM; Bedaquiline and Atovaquone = 10 μM). **b** MTT growth curve illustrating the dose-dependent synergy of ATO (0.5 μM, 1 μM, and 2 μM) with varying concentrations of ART on U937 cells treated for 48 h (*n* = 3). **c** Viability of APL (Dark green: NB4, NB4 EV-AsR1), AML cell lines (Dark blue: U937, THP-1, MV411, Kasumi-1), ALL (Light green: SUP B-15, MOLT-4 and Jurkat e6.1) and normal PBMNC and CD34 positive cells (Red; *p* < 0.05) treated with a combination of ATO and ART for 48 h. Results were normalized to their respective control. For values < 50% (dotted line), *p* < 0.0001; (normal cells *n* = 3; cell lines *n* = 7 [myeloid-4, lymphoid-3]; ATO = 2 μM; ART = 5 μM). **d** Viability of U937 cells with sequential (ATO or ART as single agents for 24 h followed by ART or ATO) and combination treatment of ATO and ART for 48 h (*n* = 3; ATO = 2 μM; ART = 5 μM). **e** Viability of U937 cells to ATO and ART in the presence and absence of glucose and glutamine(Q) (*n* = 3; ATO = 2 μM; ART = 5 μM). Proton leak, ATP production (**f**), and coupling efficiency (**g**) of U937 cells treated with ATO and ART followed by seahorse mito stress analysis (*n* = 3; ATO = 2 μM; ART= 5 μM). Viability after ATO and ART treatment for 48 h; Primary AML (**h**), B-ALL (**i**), and T-ALL (**j**) cells AML (*n* = 28), B-ALL (*n* = 24), and T-ALL (*n* = 7). ATO was used at 2 μM, and ART was at 5 μM and 20 μM. (Independent biological replicates on the freshly isolated bone marrow specimens). Data are presented as mean ± SEM. n.s., *P* > 0.05; *P < 0.05; ***P < 0.001; ***P < 0.0001, with a two-tailed unpaired t-test or one-way analysis of variance.

We had previously reported that the combination of ATO (as a glycolytic inhibitor) and non-cell-specific mitochondrial uncoupler (FCCP- Carbonyl cyanide-p-trifluoromethoxyphenylhydrazone) could re-sensitize the innate and acquired ATO-resistant cells [12]. We decided to take the leads from that study further by screening for molecules that, unlike FCCP, would specifically target leukemic cells and spare normal cells. In the current study, we evaluated the combination of ATO (2 μM) with different peroxidic antimalarials (5 μM) and anti-metabolites (5 μM) based on the available leads in the published literature [13] and focused on FDA-approved molecules that could potentially be re-purposed. Artesunate, one of the anti-malarial agents, synergized with ATO in a dose-dependent manner and elicited a profound anti-leukemic effect on U937 cells (Fig. 1a, b). The synergistic combination maintained efficacy on AML, APL, ALL cell lines, and their subtypes, with minimal perturbations on normal HSC and PBMNC (Fig. 1c). The combination did not affect the colony-forming ability of the normal haemopoietic stem cells (Fig. S1a-b). Giemsa-stained U937 cells treated with ATO or ART showed no differentiation (Fig. S1c), and caspase activation revealed that the AML cells undergo an intrinsic apoptotic pathway (Fig. S1d). We noted that concurrent treatment with ATO + ART was superior to the sequential single agents of ATO, followed by ART or vice versa (Fig. 1d).

To strengthen our dual approach targeting the leukemic cells, we evaluated the effects of ATO and ART on AML cells cultured in glutamine and glucose-free conditions. As hypothesized, ART as a single agent had a profound impact in glucose-free conditions, equivalent to the combination of ATO and ART in the presence of glucose and glutamine (Fig. 1e). We also assessed the combination of GLS (glutaminase inhibitor- CB839) and etomoxir (Fatty acid oxidation inhibitor) with ATO or ART. These agents had no synergistic effect with ATO or ART (Fig. S1e). However, we observed that ART increased GLUT-1, GLS, and CD36 expression, whereas ATO decreased GLUT-1 and did not alter CD36 and GLS expression (Fig. S1g, h). Mito stress analysis of U937 cells pre-treated with ATO and ART revealed that only ART increased proton leak, decreased ATP production, and decreased coupling efficiency (Fig. 1f, g). Measurement of reactive oxygen species (ROS) levels showed a time-dependent effect where there were no significant changes at 6 h, whereas, at 24 h, there was a reduction in the treatment groups compared to the control U937 cells (Fig S1i-j).

We then validated the therapeutic effect of ATO + ART on primary bone marrow samples of AML, BCP-ALL, and T-ALL obtained at diagnosis. We observed a dose-dependent anti-leukemic activity across the samples (Fig. 1h, j).

### Cellular targets of artesunate in AML

**Fig. 2:**
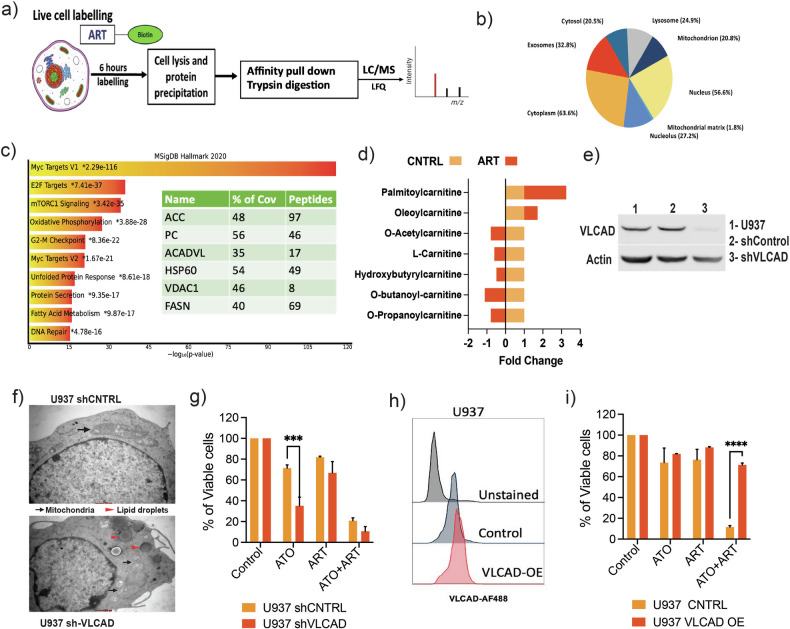
Cellular targets of artesunate in AML cells. **a** General workflow of the chemical proteomics approach where the leukemic cells are treated with biotinylated ART (50 μM, 6 h) and streptavidin pull-down followed by mass spectrometry. **b** Pie chart illustrating the cellular component enrichment from gene ontology (GO) analysis of identified direct protein targets. **c** KEGG pathway enrichment analysis of metabolites downregulated by ART in the U937 cells. (x-axis; enrichment ratio of the KEGG pathway and the intensity of red indicates the *p*-value highest to lowest). Pathway analysis of the protein targets and a table of proteins with the highest overall coverage. ACC, acetyl-CoA carboxylase 1; PC, pyruvate carboxylase; ACADVL, very long-chain specific acyl-CoA dehydrogenase; HSP60, heat shock protein 60 kDa; VDAC1, voltage-dependent anion-selective channel protein 1; FASN, Fatty acid synthase N. % Cov, percent protein sequence coverage with the identified peptides. **d** Fold change (FC) in the acylcarnitines levels identified in the untargeted metabolomics of U937 cells treated with ART (5 μM) for 24 h. **e** Immunoblot of VLCAD in U937 cells transduced with scrambled control shRNA and shRNA against VLCAD. **f** Representative electron micrographs of shVLCAD and shCNTRL U937 cells. Scale bars represent 0.5 μm. **g** Viability of U937 shCNTRL and shVLCAD cells treated with ATO and ART for 48 h (cultured in complete media) (*n* = 4; ATO = 2 μM; ART = 5 μM). **h** Flow cytometry histograms illustrate the cellular expression levels of VLCAD in the U937 cells transduced with a plasmid encoding VLCAD cDNA and its respective control. (OE: Overexpression). **i** Viability of U937 CNTRL and VLCAD OE cells treated with ATO and ART for 48 h (cultured in complete media) (*n* = 3; ATO = 2 μM; ART = 5 μM). Data are presented as mean ± SEM. n.s., *P* > 0.05; *P < 0.05; ***P < 0.001, ***P < 0.0001, with a two-tailed unpaired t-test or one-way analysis of variance.

To identify the cellular targets of ART, we employed chemical drug proteomics to label ART-interacting proteins. U937 cells (myeloid lineage, pro-monocytic) were treated with biotinylated ART (having demonstrated similar activity to the parent compound) for 6 h, and the ART targets were affinity purified by streptavidin beads and identified with LC/MS (Fig. 2a). Gene ontology analysis for cellular component enrichment showed that ART interacts with many cellular components (Fig. 2b). Pathway analysis of the enriched proteins revealed that they are involved in c-myc, OXPHOS, unfolded protein response, and fatty acid metabolism (Fig. 2c, Supplementary Table 1). We assessed the metabolic perturbation caused by ART by performing untargeted metabolomics on the U937 cells treated with ART for 24 h; we noted that acylcarnitines, triglycerides, lysophosphatidylcholine, and ceramide-related metabolites are dysregulated in comparison to control (Fig. 2d, S2, Supplementary Table 2). ART treatment significantly increased the levels of very long-chain fatty acids (VLCFA) such as palmitoyl carnitine(C16) and reduced levels of acetylcarnitine, propionyl carnitine, and L carnitine (Fig. 2d). It is known that deficiency of very long-chain fatty acid dehydrogenase (VLCAD) results in the accumulation of palmitoyl carnitine and a decrease in medium and short-chain acylcarnitine. Given that genetic knockdown (KD) and chemical inhibition (AYNE) of VLCAD promoted cell death in AML cells [18], we anticipated that ART inhibits the VLCAD enzymatic activity, as evidenced by the direct interaction from the chemical drug proteomics (Fig. 2c) and accumulation of C16 palmitoyl carnitine from our metabolomics data (Fig. 2d).

Furthermore, to corroborate our observation, we performed a KD of VLCAD in the U937 cells (Fig. 2e). We then assessed the mitochondrial changes using transmission electron microscopy and observed that lacking VLCAD altered the mitochondrial structure (Fig. 2f). As the VLCAD knockdown increases glycolysis in AML cells as a compensatory mechanism, we observed that U937 shVLCAD cells were sensitive to ATO compared to control U937 cells (Fig. 2g). Furthermore, overexpression of VLCAD in the U937 cells diminished the efficacy of the combination treatment, further confirming that the inhibition of VLCAD is crucial for the observed activity of ATO and ART (Fig. 2h, i).

### Mitophagy diminishes ART sensitivity

**Fig. 3:**
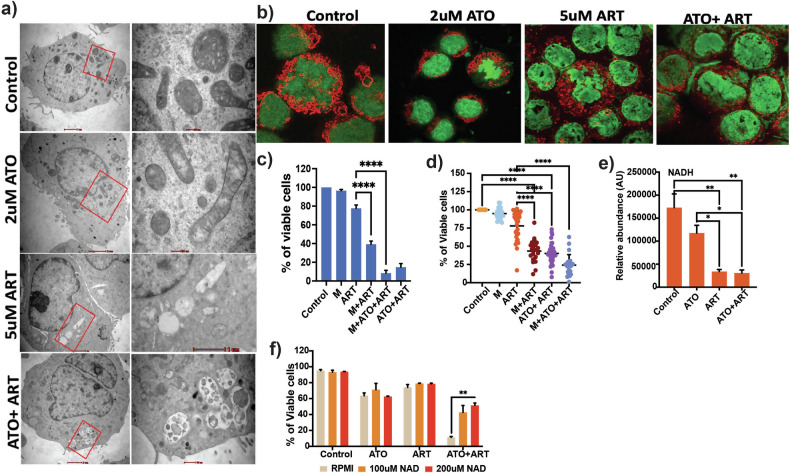
ART disrupts the mitochondrial dynamics and architecture of AML cells. **a** Representative electron micrographs showing the mitochondria morphology of U937 treated with 2 μM ATO, 5 μM and ATO + ART or control for 24 h. Scale bars represent 2 μm. **b** Representative confocal microscopic images showing the mitochondria (stained by mitotracker red CMxROS) of U937 cells treated with ATO, ART and ATO + ART or control for 24 h. Stained cells were visualized by FV3000 Inverted Confocal Laser Scanning Microscope, 63× oil immersion objective). **c** Viability of U937 cells treated with mitochondrial fission inhibitor (mdivi-1), ART, ATO, and combination for 48 h (*n* = 5; ATO = 1 μM; ART = 5 μM; mdivi-1 = 10 μM). **d** Viability of primary AML cells treated with mitochondrial fission inhibitor (MDiv-1), ART, ATO, and combination for 48 h (*n* = 22; ATO = 2 μM; ART = 5 μM; *M* = 10 μM). (Independent biological replicates on the freshly isolated bone marrow specimens). **e** LC-MS analysis of NADH levels in U937 cells treated with ATO, ART and ATO + ART for 24 h (*n* = 3; ATO = 2 μM; ART = 5 μM). **f** Viability of U937 cells treated with ATO, ART and ATO + ART when supplemented with NAD^+^ for 48 h (*n* = 3; ATO =2 μM; ART = 5 μM; NAD^+^ = 100 μM and 200 μM). Data are presented as mean ± SEM. n.s., *P* > 0.05; *P < 0.05; ***P < 0.001; ***P < 0.0001, with a two-tailed unpaired t-test or one-way analysis of variance.

ART-treated U937 cells showed disruption of mitochondrial structures with prominent loss of cristae and fragmentation visualized under the electron microscope (Fig. 3a) and confocal microscopy (Fig. 3b). The structural changes of the mitochondria suggest that the damaged mitochondria are probably actively cleared in response to ART-induced stress via mitophagy. Treatment of U937 and primary AML cell lines with ART combined with a mitochondrial fission inhibitor, mdivi-1 (mitochondrial division inhibitor 1), increased the efficacy of ART (Fig. 3c, d).

NAD^+^ pool is critical for CEM by enabling the cells to adapt to nutrient perturbations and maintain redox states. In our metabolomics data, we noted that NADH levels were reduced by the treatment of ART (Fig. 3e). Further, we assessed whether increasing the intracellular NAD^+^ would reduce the efficacy of ATO + ART. As expected, we observed that supplementation of NAD^+^ partially reduced the efficacy of the combination in U937 cells (Fig. 3f). Collectively, these results demonstrate that ART promotes uncoupled respiration and mitochondrial damage, and in combination with ATO, it inhibits compensatory glycolysis and promotes cell death.

### Intracellular iron dictates the sensitivity to ATO + ART

**Fig. 4:**
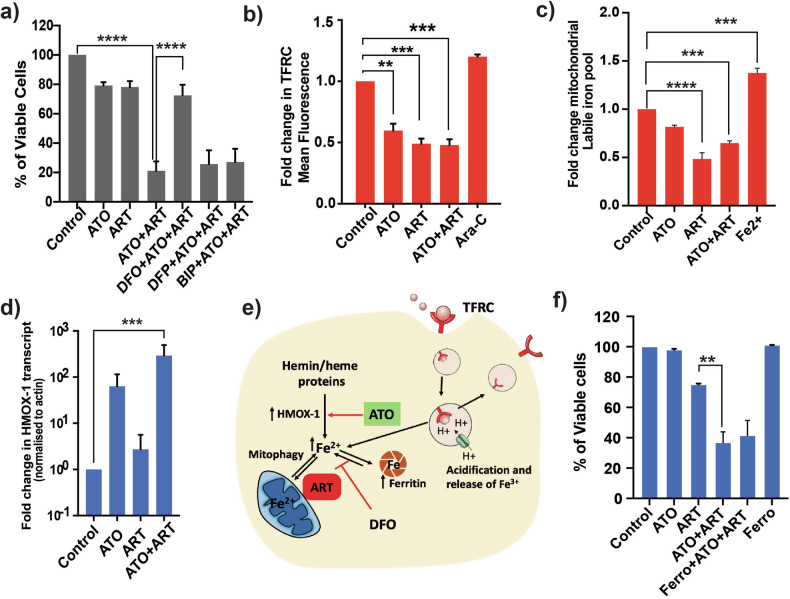
Cellular iron reserves are critical for the anti-leukemic activity of ART in combination with ATO. **a** Viability of U937 cells treated with ATO, ART, and iron chelators for 48 h (*n* = 4; ATO = 2μM; ART = 5 μM; DFO, DFP, and BIP = 2 μM). **b** Fold change in transferrin receptor (TFRC) surface expression mean fluorescence intensity on U937 cells treated with ATO, ART, ATO + ART, and cytarabine (Ara-C) for 24 h (*n* = 3; ATO = 2 μM; ART = 5 μM; ARA-C = 400 ng). **c** Fold change in mean fluorescence of MitoFerro green -1 dye intensity that explicitly measures the mitochondrial liable pool levels in U937 cells treated with ATO, ART, and ATO + ART, Fe^2+^ as positive control (*n* = 3; ATO = 2 μM; ART = 5 μM). **d** Transcript levels of haem oxygenase-1 on U937 cells treated with ATO and ART for 24 h (*n* = 6; ATO = 2 μM; ART = 5 μM). **e** Schematic showing the labile iron pool in leukemic cells and the effect of ATO and ART. ART targets the mitochondria and diminishes the iron pool by utilising for its activation. TFRC mediated iron uptake and lysosomal release of iron, Heme-based iron supplementation, and degradation of Ferritin also contributes to iron source for enhanced ART activation. ATO increases the hemin/heme containing degrading enzyme HMOX-1 increasing the iron availability for ART. DFO—an iron chelator, reduced the availability of iron for ART thereby limiting its anti-leukemic activity. (Created with Adobe Illustrator). **f** Viability of U937 cells treated with ferroptosis inhibitor (ferrostatin1–Ferro 1) alone and in combination with ATO and ART for 48 h (*n* = 3; ATO = 1 μM; ART = 5 μM; Ferrostatin-1 (ferro = 10 μM)). Data are presented as mean ± SEM. n.s., *P* > 0.05; *P < 0.05; ***P < 0.001; ***P < 0.0001, with a two-tailed unpaired t-test or one-way analysis of variance.

Iron plays a vital role in the antimalarial activity of artemisinins by catalyzing activation of the endoperoxide moiety. It is recognized in leukemias that the leukemic cells have enhanced iron uptake via transferrin receptor (TFRC), decreased ferroportin (efflux transporter), and increased cellular content of iron for cell proliferation [19]. It has also been noted that TFRC expression in cancer cells correlates with susceptibility to ART [20]. Deferoxamine (DFO), an iron chelator, diminished the antimalarial activity of artemisinins by chelating the intracellular iron required for their activity [21–23]. Hence, we used iron chelators that have differential affinity for different intracellular compartments, such as deferiprone (DFP; mitochondria and cytosol), DFO (lysosomes), and 2’2’ Bipyridyl (BIP; cytosol), with ATO + ART to assess the importance of subcellular compartments of iron for anti-leukemic activity. DFO significantly abrogated the anti-leukemic activity of ATO + ART, whereas DFP and BIP did not (Fig. 4a). DFO primarily chelates the lysosomal iron (iron from transferrin or ferritin via ferritinophagy or mitochondria via mitophagy), limiting the available iron pool for ART activity. The observed rescue in viability was because of DFO on the major cellular iron pool.

DFO is transported into the cell via an endocytosis process similar to the iron-loaded transferrin receptor. We then measured the cell surface expression of transferrin receptor (TFRC) as TFRC-mediated iron uptake is the major cellular iron uptake pathway. Transferrin-bound iron (holo-transferrin) is taken up via the receptor-mediated endocytosis pathway, and endosomes fuse with lysosomes, releasing iron in the cytosol and recycling receptors for apo-transferrin to the cell surface. We assessed the effect of ATO + ART on the surface expression of TFRC and labile iron in different iron compartments. ATO and ART as single agents reduced the surface expression of TFRC (Fig. 4b), indirectly modulating the intracellular iron pool. This effect was only observed with ATO and ART, not with other chemotherapeutic agents. DFO also rescued ATO-induced cell death in NB4 cells (sensitive to ATO) but did not impact cell death induced by daunorubicin or cytosine arabinoside (Ara-C) (Supplemental Fig. 3).

Notably, the measurement of the mitochondrial labile pool (Mito ferro Green-1—mitochondria-specific labile iron probe) revealed that ART reduced the availability of the labile Fe^2+^ pool compared to ATO and control cells (Fig. 4c). Cytosolic iron levels (calcein-AM) were not changed with the treatment of ATO or ART and their combination (data not shown).

We also examined the levels of haem oxygenase 1 (HMOX-1, a metabolic enzyme degrading haem-containing proteins and releasing free iron, bilirubin, and carbon monoxide), which ATO is known to increase in cancer cells [24]. ART effect has also been shown to be limited in cells that lack HMOX-1 [25]. Hence, we measured the expression of HMOX1 and, as expected, treating U937 cells with ATO, ART, and ATO + ART increased the HMOX-1 expression, supporting ART’s existing mechanism of action (Fig. 4d). Figure 4e illustrates the efficacy of this combination and the additional mechanism of synergy by the action of ATO in increasing free iron for the activity of ART. As the involvement of iron was evident in the process of cell death, we assessed whether it is a ferroptotic cell death. A combination of ferroptosis inhibitor Fer-1 (ferrostatin-1) with ATO and ART did not affect the observed cell death (Fig. 4f). These findings suggest the importance of intracellular iron in determining the activity of ATO and ART.

### Iron enhances the anti-leukemic properties of ART

**Fig. 5:**
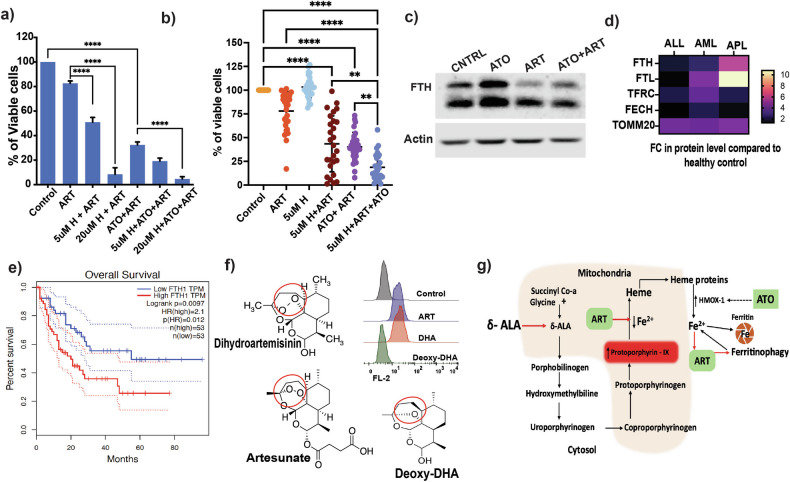
The specificity of the combination therapy is shaped by the decisive presence of intracellular iron. **a** Viability of U937 cells treated haemin (H) in combination with ART and ATO + ART for 48 h (*n* = 4; ATO = 2 μM; ART = 5 μM; Haemin (H) = 5 μM and 20 μM). **b** Viability of primary AML cells treated with haemin (H), ART, ATO, and combinations for 48 h (*n* = 27; ATO = 2 μM; ART = 5 μM; Haemin (H) = 5 μM). (Independent biological replicates on the freshly isolated bone marrow specimens). **c** Immunoblot of FTH in U937 cells post-exposure to ATO, ART and ATO + ART for 24 h (*n* = 3; ATO = 2 μM; ART = 5 μM). **d** Heatmap of TMT-labelled proteome (FTH, FTL, TFRC, FECH, TOMM20) of primary AML, APL, and ALL cells compared to healthy control mononuclear cells. (5 samples per group, and the differentially expressed proteins and fold change were calculated compared to healthy control mononuclear cells). **e** Kaplan-Meier curves of overall survival in TCGA AML patients in the FTH-high red (red) and FTH-low (blue) groups. **f** Structure highlighting the endoperoxide moiety of artesunate (ART), active metabolite dihydroartemisinin (DHA), and the inactive metabolite deoxy dihydroartemisinin (Deoxy DHA). Histograms illustrating FL2 shift on flowcytometry in the U937 cells treated with these three agents for 24 h and the fluorescent intermediates that accumulate as a result with ART and DHA but not with Deoxy-DHA (*n* = 3; 5 μM = ART, DHA, and Deoxy derivative). **g** Protoporphyrin –IX (PPIX) is a fluorescent intermediate of the heme biosynthesis pathway. The precursor of heme biosynthesis, a-ALA, enhanced ART activity, as seen in heme-based supplementation. The ART utilization of iron results in less/lack of iron for the production of heme, which, in turn, leads to the accumulation of fluorescent intermediate PPIX that is observed with ART and its active intermediate DHA, not with the deoxy form ART/DHA. Hemin/Heme containing degrading enzyme HMOX-1 and ferritin is increased by ATO, thereby increasing the availability of iron for ART activity (Created with Adobe Illustrator). Data are presented as mean ± SEM. n.s., *P* > 0.05; *P < 0.05; ***P < 0.001; ***P < 0.0001, with a two-tailed unpaired t-test or one-way analysis of variance.

To characterize in detail how iron enhances the activity of ART, we treated U937 cells with transferrin (holo and apo forms), Iron II sucrose, hemin (protoporphyrin IX containing Fe^3+^) and δ-aminolaevulinic acid (ALA). We noted that only heme-based iron supplementation (using hemin and ALA predominantly in the mitochondria) showed synergy in enhancing the apoptotic activities of ART and ATO + ART (Fig. 5a, Supplemental Fig. 4a, b). We observed similar effects on primary bone marrow specimens of AML (Fig. 5b). We examined the intracellular iron storage protein ferritin levels, which would be degraded when needed to meet the cellular iron demands. We observed that ATO enhances the intracellular iron reserve and storage, which is evident by the increase in FTH levels (Fig. 5c). In contrast, ART, which requires iron for the endoperoxide activation, utilizes the iron reserve and promotes cell death, which further corroborates the rescue observed with the iron chelator DFO and the importance of iron in their synergism.

Our published TMT-labelled proteomics of primary leukemia samples compared to normal peripheral blood cells showed that the leukemic cells had increased iron-related proteins (FTH, FTL, TFRC, FECH, and TOM20) (Fig. 5d). To assess clinical relevance, we interrogated the Cancer genome ATLAS data of AML and noted that higher expression of FTH at diagnosis predicted poorer overall survival (Fig. 5e). The endoperoxide moiety of the artemisinin family plays a vital role in the antimalarial and anti-leukemic activity of ART and its active metabolite dihydroartemisinin (DHA). The endoperoxide moiety reacts with iron and heme, sequestering it and resulting in an iron-deprived environment, as evidenced by the accumulation of protoporphyrin IX (PpIX – a fluorescent metabolite) [26, 27].

To confirm this, we tested a deoxy derivative of artemisinin lacking the reactive endoperoxide ring that is reactive to iron and heme. The leukemic cells treated with the deoxy derivative did not produce the fluorescence seen in ART and DHA (Fig. 5f). These observations are summarized in the illustration Fig. 5g. The absence of fluorescence in normal MNC’s treated with ART compared to AML cells (data not shown) also illustrates the relative specificity of the effect of ART on leukemia cells, linked to their intrinsically altered handling of iron and higher levels of intracellular iron.

### Novel ART-based combinations are effective in AML

**Fig. 6:**
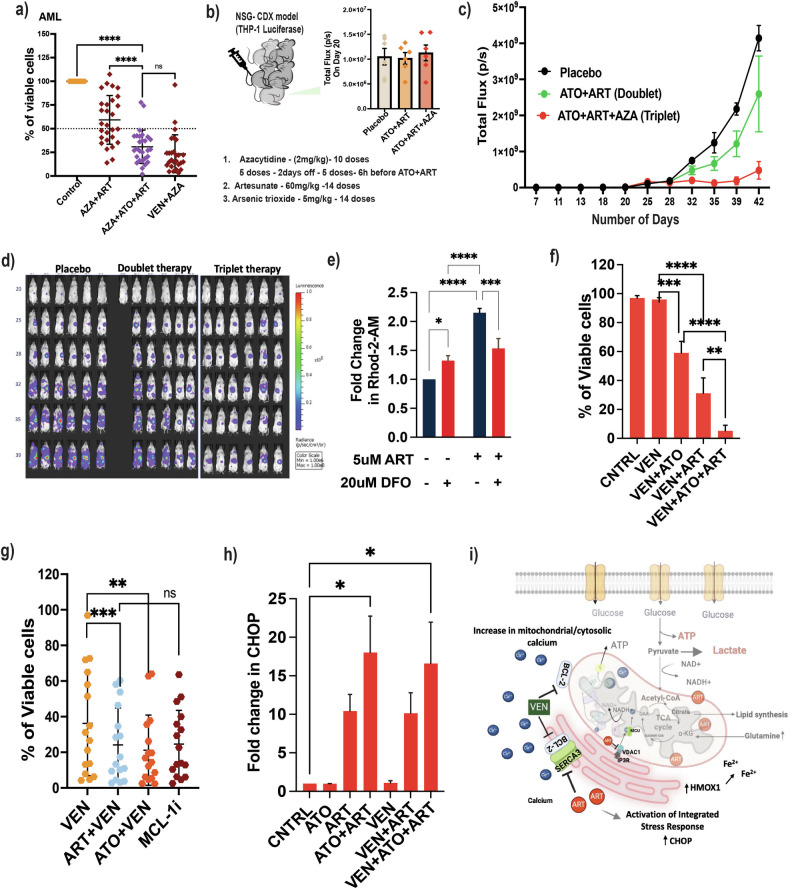
Novel ART-based combinations are effective in AML. **a** Viability of primary BM AML samples treated with VEN, ART, ATO, AZA, and rationale combinations for 48 h (*n* = 28; ATO = 2 μM; ART = 5 μM; VEN = 500 nM; AZA = 2.5 μM). (Independent biological replicates on the freshly isolated bone marrow specimens). **b** THP-1 luciferase cell line-derived xenograft model and the leukemic burden before initiation of combination therapies (6 animals per experimental group). **c** The bioluminescent intensity of photons emitted from each mouse in the images was quantified during the experiment. Tumour growth was monitored every 4 days by bioluminescence imaging in THP-1 luciferase-engrafted mice and the experimental groups (Black—placebo; Green—ATO + ART, and Red—ATO + ART + AZA). **d** Bioluminescent images of mice transplanted with THP-1 luciferase cells. Mice were administrated with a vehicle, ART + ATO, and ATO + ART + AZA from day 21 to 34 post-transplantation, as described in Fig. 6b. The same mice are depicted at each time point (*n* = 6 mice per group). **e** Mitochondrial calcium levels of U937 cells treated with ART in the presence and absence of an iron chelator (DFO) for 24 h. Measured using mitochondrial calcium-specific dye Rhod-2 AM. (*n* = 3; ART = 5 μM; DFO = 20 μM). **f** Viability of U937 cells treated with VEN, ART, and ATO for 48 h (*n* = 3; ATO = 2 μM; ART = 5 μM and VEN = 500 nM). **g** Viability of primary AML cells treated with VEN in combination with ART and ATO, and MCLi inhibitor (*n* = 16; ATO = 2 μM; ART = 5 μM; VEN = 500 nM; MCLi = 100 nM) for 48 h. (Independent biological replicates on the freshly isolated bone marrow specimens). **h** Transcript level of CHOP; ER stress response gene post 24 h treatment with ART in combination with ATO, VEN, and ATO + ART + VEN (*n* = 3; ATO = 2 μM; ART = 5 μM; VEN = 500 nM). **i** *Hypothetical model depicting the synergy of ART and VEN*: ART’s known anti-malarial target SERCA3. BCL-2 interacts with the SERCA3 Ca2+ pump and regulates Ca2+ mobilization to the endoplasmic reticulum. VEN inhibits the BCL2, thereby affecting the Ca2+ homeostasis, whereas ART directly targets SERCA3 activity, depleting ER calcium reserves and increasing the cytosolic and mitochondrial Ca2+. The dysregulation of the cellular Ca2+ and iron imbalance promotes energy crisis and activation of the final integrated stress response common pathway (Created with biorender.com). Data are presented as mean ± SEM.n.s., *P* > 0.05; *P < 0.05; ***P < 0.001; ***P < 0.0001, with a two-tailed unpaired t-test or one-way analysis of variance.

Azacytidine (AZA), combined with venetoclax, is used as front-line treatment for elderly patients and those with a poor performance score who are considered unfit to receive standard intensive induction therapy. We evaluated ATO + ART with AZA to assess the potential role of this clinically relevant combination on primary AML samples and confirmed that these drug combinations are not antagonistic (Fig. 6a). The triple combination (ATO + ART + AZA) had minimal effect on the normal peripheral blood mononuclear cells (Fig. S5). We evaluated the in vivo efficacy of ATO + ART and ATO + ART + AZA in a THP-1 AML cell line-derived luciferase xenograft model (Fig. 6b). We noted a significant reduction in the overall leukemia burden with ATO + ART and ATO + ART + AZA (Fig. 6c, d).

ART is known to have an inhibitory effect on SERCA3 [28, 29]. We questioned whether it impacted the AML cells’ mitochondrial Ca²⁺ levels, since both mitochondrial calcium overload and insufficiency have been shown to suppress OXPHOS [16, 30, 31]. Measurements of mitochondrial Ca²⁺ in U937 cells treated with ART showed a significant increase. To further understand its relation to iron, we measured the mitochondrial Ca^2+^ in the presence of DFO. Despite the presence of DFO, the ART-induced elevation in mitochondrial Ca2+ persisted, albeit at a reduced level, highlighting the role of iron in the ART-induced increase in Ca^2+^ levels (Fig. 6e). This suggests that elevated mitochondrial Ca2+ could be due to both ART activity on depletion of mitochondrial iron and SERCA3 inhibition. Further analysis of SERCA3 and SERCA2 expression across the TCGA tumor data revealed that the AML cells had increased SERCA3 expression compared to normal. The SERCA3 to SERCA2 expression ratio across the tumor samples was significantly high in DLBCL, AML, and thymoma (Supplemental Fig. 6a-b). High SERCA3 (ATP2A3) expression in the TCGA LAML cohort predicted poor survival outcomes (Supplemental Fig. 6c).

Recent understanding of the VEN mechanism of action beyond BCL-2 [32] prompted us to assess whether the combination of ART can overcome VEN resistance. A combination of ART and/or ATO synergized with VEN and promoted cell death in both the VEN-resistant AML cell line (U937) and primary human AML specimens (Fig. 6f, g). The observed effect of VEN + ART and VEN + ATO in primary human AML specimens was equivalent to that seen with single-agent MCLi inhibition (S63845).

Our findings hypothesize a final common pathway for novel ART-based combinations through an increased integrated stress response (ISR) and subsequent derangement of mitochondrial iron and calcium homeostasis. To assess ART’s effect on ISR, we measured CHOP levels. ART significantly increased CHOP transcription in AML cells, and the effect was further enhanced by combining it with ATO and VEN. Neither VEN nor ATO alone significantly impacted CHOP transcript levels (Fig. 6h).

Based on these observations, we propose a model where ART promotes mitochondrial dysfunction (uncoupled respiration and structural changes) by utilizing mitochondrial iron and mitochondrial calcium overload in AML, leading to an increase in ISR (Fig. 6i) and activation of the intrinsic apoptotic pathway.

## Discussion

Affordable and safe treatments for AML and those that can overcome resistance to conventional treatment regimens are urgently needed. With the advent of targeted therapies, there remains a need to define the optimal dosing, schedule, and efficacious combinations with minimal off-target effects. Using a combination of ATO and ART, we have demonstrated a drug repurposing strategy that targets a metabolic vulnerability of AML and could be combined with standard therapy to enhance efficacy without significant off-target side effects or toxicity that was observed with complex-I inhibitor IACS-010759 in the Phase I clinical trials [33, 34]. ART is a very well-tolerated and safe antimalarial that is being developed for additional antiparasitic, antiviral, and other anticancer indications. ART has many targets in different systems, including SERCAs in malarial and mammalian cells [23]. ART also targets mitochondria, enhances mitophagy [35], promotes cytochrome-c release [36], and interferes with many master regulatory pathways in cancer models [37–39].

We validated that ART acts as a cancer cell-specific mitochondrial uncoupler. AML cells enhance their glycolytic potential to survive OXPHOS inhibition; this is a common escape mechanism that has contributed to the failure of a therapeutic benefit in previous efforts at OXPHOS inhibition and has also contributed to the increased toxicity. ATO has been shown to inhibit several key glycolytic enzymes and the pyruvate dehydrogenase complex [40, 41]. We have demonstrated that combining ATO with ART abolishes the metabolic switch and disturbs the central energy metabolism, specifically in the leukemic cells. Chemical drug proteomics revealed that ART has pleiotropic targets involved in fatty acid synthesis, degradation, and mitochondrial pathways, consistent with previous studies in solid tumors and malarial parasites [42–44]. Mitochondrial proteins such as CLPB (caseinolytic peptidase B) and VLCAD [18, 45], which have been shown as potential therapeutic targets in AML cells, were also labeled by ART. We further demonstrated that ART mimics the effects of inhibition of VLCAD in vitro and knockdown of VLCAD in AML cells synergized with ATO and promoted cell death. Genetic inhibition of VLCAD has been shown to increase PDH activity and glycolysis in AML cells, and the sensitivity of these cells to single-agent ATO corroborates our findings.

ART treatment disrupted mitochondrial cristae in AML cells and promoted mitophagy. The combination of a mitophagy inhibitor enhanced cell death even in the absence of ATO, demonstrating that cells with an active mitophagic process would be resistant to or less sensitive to ART. Similarly, cellular pools of NAD^+^/NADH regulate OXPHOS, and an increased NAD^+^ pool has been reported to be associated with relapse [46]. The availability of NAD+ is critical for NAD-dependent enzymes in the TCA cycle and glycolysis [47]. Supplementation of NAD^+^ partially reduced the anti-leukemic efficacy of ATO + ART.

Intracellular iron plays a critical role in the efficacy of ART [48], particularly in combination with ATO against AML. This combination leverages the distinct iron metabolism of malignant cells, which was illustrated by the lack of their detrimental effects on normal cells and in the presence of an iron chelator (DFO). Beyond its impact on the glycolytic inhibition, ATO increases the intracellular iron pool via HMOX-1 and reserves, which is a critical need for ART. Supplementation with iron, for instance, as ALA or haemin, significantly boosted ART’s antileukemic properties even in the absence of ATO, underscoring the critical dependence of ART’s efficacy on cellular iron levels. ART’s interaction with labile iron led to the accumulation of fluorescent intermediates of heme biosynthesis. Notably, deoxyDHA, lacking the endoperoxide bridge that is reactive to iron, fails to induce this accumulation, reinforcing the central role of iron.

Moreover, leukemic stem cells (LSCs) rely on ferritin degradation via ferritinophagy to maintain their iron bioavailability, highlighting this pathway as a potential therapeutic vulnerability [49]. In summary, the heightened expression of TFRC, increased FTH, and elevated intracellular iron in leukemic cells contribute to the relative specificity of the ART + ATO combination against malignant cells, sparing normal counterparts. This combination has demonstrated significant antileukemic activity in acute lymphoblastic leukemia, suggesting its broad applicability to other leukemia types and tumor models characterized by robust mitochondrial activity and altered iron metabolism.

VEN + AZA is the favored induction therapy for elderly patients who cannot tolerate 7 + 3 induction [50]. However, resistance to VEN can be inherent in a subset of AML or acquired during the treatment, by switching the reliance on alternative mitochondrial anti-apoptotic proteins such as MCL1. BCL-2 has been identified to interact with SERCAs, and this interaction regulates the function of SERCA3, which has been reported to be overexpressed in VEN-resistant AML patients [32]. Interaction of BCL-2 family proteins (BCL-2 and BCL-XL) with SERCA and VDAC calcium pumps regulates the flux and transfer of calcium across the membranes and organelles [51, 52]. Both VEN, by its action on BCL-2, and ART, via its action on SERCA-3, lead to mitochondrial dysfunction and apoptosis of AML cells. Given the complementary mechanisms, combining ART with ATO, potentially alongside standard therapies such as AZA or VEN, offers a strong rationale for these combinatorial approaches with minimal off-target side effects on normal cells. Clinical trials are warranted to define these novel drugs’ optimal combinations, doses, and schedules to achieve their potential.

## Methods

### Cell culture experiments

All cells were grown at 37 °C in a 5% CO2 humid atmosphere. NB4 (a kind gift from Dr. Harry Iland, RPAH, Sydney, Australia, with permission from Dr. Michel Lanotte) (RRID:CVCL_0005), NB4 EV-AsR1, NB4 EV-AsR2 (in-house generated ATO resistant cell line), MV-411 (ATCC), THP-1 (ATCC), U937 (ATCC) (RRID:CVCL_0007), and Jurkat e6.1(ATCC) (RRID:CVCL_0367)cells were cultured in RPMI-1640 medium (Sigma) containing 2 mM L-glutamine, supplemented with 10% fetal bovine serum (ThermoFisher Scientific) and 1× penicillin-streptomycin (ThermoFisher Scientific). U937 was further confirmed by STR profiling. Kasumi-1 (ATCC) (RRID: CVCL_0589) cells were cultured in RPMI-1640 medium containing 2 mM L-glutamine, supplemented with 20% fetal bovine serum and 1× penicillin-streptomycin. SUP B15 (ATCC) cells were cultured in Iscove’s Modified Dulbecco’s culture medium (Sigma) supplemented with 20% fetal bovine serum and 1× penicillin-streptomycin (ThermoFisher Scientific). Cell lines were tested for mycoplasma contamination every six months in the laboratory using a Universal Mycoplasma detection kit (ATCC).

### Primary human Specimens

Primary Samples were collected after informed consent was obtained, and studies were conducted under the protocols approved by the Institutional Research Ethics Committee (IRB Min No -11796). Consecutive bone marrow specimens from patients diagnosed as AML, ALL, and APL who were willing to consent to participate were included in the study (Feb 2020–Jun 2021). Mononuclear cells were isolated using a Ficoll density gradient. The primary samples were cultured in Iscove’s modified Dulbecco’s culture medium (Sigma) supplemented with 20% fetal bovine serum and 1× penicillin-streptomycin (ThermoFisher Scientific).

### Animal studies

NSG (NOD.Cg-PrkdcscidII2rgtm1wjl/SzJ) was purchased from Charles River Laboratories. 10^6^ THP-1 luciferase viable cells were transplanted into 10 to 12-week-old male mice by intravenous injection. Tumour growth was checked twice weekly (from day 7) by bioluminescent imaging (BLI). Treatment commenced once progressive tumor growth was evident (Day 20), and randomization was based on bioluminescence. The drugs were administered intraperitoneally, and the treatment continued for two weeks with an 11-day observational phase followed by a further five days of dosing. Bioluminescence measurements were performed every 4 days to monitor leukemic burden. The Crown Biosciences United Kingdom performed the experiments according to their CBUK guidelines and ethical approval (Study number: CSU4381).

### Cell death assays

0.5 M cells were treated with test compounds for 48 h in the respective growth media. Post-treatment viability was measured using an Annexin V/7AAD apoptosis assay kit (BD Pharmingen). Flow cytometry data was analyzed using FlowJo version 8 (RRID:SCR_008520). Viability was calculated as a percentage of cells negative for annexin-V and 7AAD. For the dose-response curve, 10^5^ cells per well in a 96-well plate were treated with increasing concentrations of ART in combination with ATO. 10ul of MTT solution (5 mg/ml) was added to the wells after 48 h of treatment the plate was incubated for 4 h and the formazan crystals formed were dissolved in 10% acidified sodium dodecyl sulphate. The absorbance was read at 570 nm with a reference wavelength of 630 nm (Spectramax M4). The data were normalized to the respective control.

### Seahorse metabolic flux analysis

Mito stress XF24 assay kit (Agilent Technologies, CA, USA) measured oxygen consumption. 5 × 10^5^ cells were plated in an XF24 cell culture microplate. Oxygen consumption and glycolytic flux were measured according to the manufacturer’s protocol and as previously described [12]. 5 × 10^5^ cells per well were seeded in a 24-well XF24 plate. Thirty minutes before analysis, the medium was replaced with mito stress media (Agilent Technologies), the plate was incubated at 37 °C non-co2, and a mito stress test was performed. Analyses were performed at basal conditions and after oligomycin, FCCP, antimycin A, and rotenone injection.

### Chemical-drug proteomics approach for target identification

Biotinylated-artesunate (B-ART) was synthesized by linking PEG-Biotin to the dihydroartemisinin (the active metabolite of artesunate) and used to pull down the interacting proteins and streptavidin on bead digestion were carried out. U937 cells were treated with B-ART for 6 h in combination with ATO at the end of incubation; the cells were lysed using MS-compatible lysis buffer (50 mM Tris (pH 8.0), 150 mM NaCl, Protease inhibitors) and clear supernatant were collected and proceeded with streptavidin pull down to enrich biotinylated proteins as per the manufacturer’s protocol (♯Thermofisher scientific). On column trypsin digestion were performed on the samples. Non-biotinylated control samples were used to identify and exclude non-specific targets of biotinylated ART. Peptides were identified and quantified using protein pilot software 4.5(SCIEX) with a paragon algorithm in reference to the International Protein Index (IPI database).

### Mitochondrial morphology using confocal laser scanning microscopy

MitoTracker™ Red CM-H2Xros (Thermo FisherScientific) was added into culture media at a final concentration of 100 nM to label the mitochondria of leukemic cells for 30 min in 37 °C 5% CO2 incubator before harvesting cells for confocal studies. To prepare slides, labeled cells were washed 2 times with ice-cold PBS and re-suspended at a final concentration of 500 K cells /ml in ice-cold PBS. At room temperature, 150 µl of cell suspension (75 K cells) was used for cytospin at 500 rpm for 5 min. The slides were air-dried in the dark box at room temperature for 5 min before being fixed in −20 °C 100% methanol for 10 min. The slides were then air-dried in a dark box at room temperature for 10 min and mounted in Vectashield DAPI mountant before confocal imaging. The stained slides were imaged on the Olympus FV3000 confocal microscope using its high-sensitivity detectors to excite the DAPI and its 633 nm laser. Z stacks were captured, and the images were deconvoluted using Olympus Fluoview.

### Measurement of mitochondrial Labile iron pool

Briefly, 5 × 10^5^ cells were treated with drugs and after 6 h, the cells were stained with Mito Ferro green-1 (Dojindo Molecular Technologies, Inc.) for 15 min in Phosphate buffered saline (PBS) at 37 °C followed by washing with PBS and the fluorescence intensity was measured in FACS Calibur with an excitation wavelength at 485 nm, and the data analyzed using Kaluza Analysis Software (Beckman Coulter).

### Transmission electron microscopy

Two million leukemic cells were collected post-24 h of treatment or control and washed twice with phosphate buffer. The cells were then fixed with a fixative -4% paraformaldehyde plus 3% glutaraldehyde in 0.1 M phosphate buffer pH 7.2 (PB)-for 30 min at room temperature and 4 °C overnight. Cells were then washed with phosphate buffer three times for 15 min. Next, cells were post-fixed with 1% osmium tetroxide buffered with PB for 1 h, washed again using PB twice for 20 min, dehydrated with an ethanol series, and then infiltrated with propylene oxide. The samples were then resin-embedded with Epon and polymerized at 60 °C for 48 h. Solid epoxy blocks were sectioned on a Leica Ultracut microtome to 100 nm thickness. The ultra-thin sections were collected on copper grids and stained with uranyl acetate and Reynolds solution (sodium citrate and lead citrate) to give contrast. Sections were transmitted in electron microscopy Tecnai T12 spirit and photographed.

### Statistical analysis

Statistical analyses were performed using GraphPad Prism version 9.3.1 (RRID:SCR_002798). Data are presented as mean ± SEM. All data points are represented as mean ± standard error of the mean. A two-tailed Student t-test was used to compare mean values between the 2 groups. A one-way analysis and two-way variance analysis was used for experiments in which multiple groups were compared with the control group. Results are considered significant when *p < 0.05, **p < 0.01, ***p < 0.001, and ****p < 0.0001.

## Supplementary information

Supplementary Methods and Figures

Uncropped Westernblots

Supplementary 2_Biotnylated Pull down ART

Supplementary 3_DMSO_ART Metabolomics data

## Data Availability

No codes were generated or used in the study. Further information and requests for resources and reagents should be directed to and will be fulfilled by the Lead Contact, Dr. Vikram Mathews (vikram@cmcvellore.ac.in).

## Code availability

No codes were generated or used in the study. Further information and requests for resources and reagents should be directed to and will be fulfilled by the Lead Contact, Dr. Vikram Mathews (vikram@cmcvellore.ac.in).
