# Supplementary material for: A novel strategy to target metabolic dependencies in acute myeloid leukemia
Source: Cell Death Dis. 2025 Nov 4;16(1):792. doi: 10.1038/s41419-025-08129-3 (PMC12586699; doi:10.1038/s41419-025-08129-3)
Supplement: Supplementary file 1 — Supplementary Methods and Figures [file 41419_2025_8129_MOESM1_ESM.pdf]

# A Novel Strategy to Target Metabolic Dependencies in Acute Myeloid Leukemia

Nithya Balasundaram[1,2], Hamenth Kumar Palani[1], Arvind Venkatraman[1], Yolanda Augustin[3], Shruthi Pichandi[1], Clement Regnault[4], Majeela Solomon[1], Abirami Rajasekaran[1], Mohammed Yasar[1], Swathy Palani Kumar[1], Reeshma Nair Radhakrishnan[1], Anu Korula[1], Uday Prakash Kulkarni[1], Eunice Sindhuvi Edison[1], Poonkuzhali Balasubramanian[1], Biju George[1], Aby Abraham[1], Sanjeev Krishna[3, 5-7, *], Vikram Mathews[1*].

[1]Department of Haematology, Christian Medical College, Vellore, India.

[2]Laboratory of Cellular Metabolism and Microenvironment, De Duve Institute, UC Louvain, Brussels, Belgium.

[3]Clinical Academic Group in Institute for Infection & Immunity, St George's University of London, London, United Kingdom

[4]MVLS Shared Research Facilities, University of Glasgow, Glasgow, United Kingdom.

[5]St George's University Hospitals NHS Foundation Trust, United Kingdom

[6] Institut für Tropenmedizin, Universitätsklinikum Tübingen, Tübingen, Germany

[7]Centre de Recherches Médicales en Lambaréné (CERMEL), Lambaréné, Gabon

* These authors equally contributed to the work.

**Supplemental Methods:**

**Colony Formation Unit (CFU) assay:** $CD34^+$ hematopoietic stem and progenitor cells (HSPCs) were then enriched from healthy donors' mobilized peripheral blood cells through positive selection using the CD34 MicroBead Kit (Miltenyi Biotec) according to the manufacturer's protocol. Following purification, cells were resuspended in hematopoietic stem cell colony-forming unit (HSC-CFU) medium. $10^4$ CD34 positive cells, single-cell colony formation, the cell suspension was diluted and seeded into 96-well plates so each well contained a single $CD34^+$ cell. The plates were incubated at 37 °C in a humidified atmosphere with 5% $CO_2$ for a period of 14 days to allow colony formation and differentiation. After 14 days, the cells were analysed for colonies and to identify the type of colonies, the cells were stained with a lineage-specific antibody cocktail comprising CD235a (erythroid lineage), CD14 (monocytic lineage), and CD15 (granulocytic lineage). Flow cytometry analysis was performed to enumerate colonies and evaluate lineage commitment based on surface marker expression per the manufacturer's protocol (StemMACS™ HSC-CFU Assay Kit, Human).

**Semi-quantitative real-time polymerase chain reaction:** Total RNA was extracted using Trizol reagent (Invitrogen Carlsbad, CA, USA). 500ng of the extracted RNA was converted into cDNA using superscript II cDNA kit (Invitrogen Carlsbad, CA, USA). The expression of genes was studied using SYBR green method (Finnzymes F410L, Thermo scientific, Rockford, IL, USA). The Ct values were normalized with actin and the fold differences were calculated using 2-ΔΔCt method. Following primers were used: ACTIN (For: GGCGGCACCACCATGTAC CCT Rev: AGGGGCCGGACTCGTCATACT), HMOX-1 ( For: AAG CCG AGA ATG CTGTCA Rev: GCC GTG TAG ATA TGG TACAAGGA), GLUT-1 (For: CATCAATGCCCCCCA Rev: AAGCGGCCCAGGATC),GLS (For: TCTACAGGATTGCGAACGTCT Rev: CTTTGTCTAGCATGACACCATCT),CHOP(For: AGCCAAAATCAGAGCTGGAA Rev: TGGATCAGTCTGGAAAAGCA).

**Genetic knockdown and overexpression studies:** U937 cells were transduced with ACADVL shRNA lentiviral particles (SC-72427-V) and control shRNA lentiviral particles (SC-108080) and ACADVL (VLCAD) Lentiviral Activation Particles (h2) (sc-403111-LAC-2) in the presence of 8ug/ml of polybrene (Sigma Aldrich). The lentiviral particles were purchased from Santacruz biotechnology. After transduction, the cells were selected using 1 µg/ml of puromycin. The knockdown efficiency was confirmed with immunoblotting (ACADVL Rabbit pAb, abclonal A21470), and the overexpression was confirmed by flow cytometry (ACADVL Antibody (H-7) Alexa Fluor® 488 (sc-376239 AF488).

**Immunoblot:** Cell homogenates were obtained by cell lysis in IP Lysis buffer (Thermo Scientific), with complete protease inhibitors (Roche, Basel, Switzerland). Protein was quantified using Bradford assay (Bio-Rad). 25ug of lysates per lane was loaded were analysed in SDS–PAGE. After protein transfer to a nitrocellulose membrane, membranes were blocked with non-fat dry milk (5%, 2h) and incubated with primary antibodies overnight (Ferritin heavy chain (B-12) (SC-376594), (ACADVL Rabbit pAb, abclonal A21470), (Actin (8H10D10) Mouse Ab, Cell Signalling Technologies 3700S) and Caspase-3 (Rabbit, Cell Signalling Technologies 9662S). After an overnight incubation, the membranes were washed thrice with wash buffer containing Tween 20 and incubated with HRP conjugated anti-mouse and anti-rabbit secondary antibody (Cell Signalling Technologies) for an hour. The membranes were washed

5 times, and the protein bands were detected by the standard chemiluminescence method (ThermoFisher Scientific).

**Wright-Geimsa Staining:** 50K U937 cells in one hundred microliters were used to make cytospin slides (500RPM for 5 minutes). The slides were then air-dried and fixed with one hundred per cent methanol for 10 minutes, washed and stained with Wright-Giemsa for 5 minutes, and washed with PBS, and the images were captured in an inverted light microscope using an oil immersion lens (Olympus).

**Endogenous ROS measurement:** $5x10^5$ treated/control cells were stained with 5uM of DCFDA (Sigma) for 15 minutes at 37 degrees. The stained cells were washed twice with phosphate-buffered saline (PBS) at 500g for 5 minutes. The cell pellet was resuspended in PBS, and the fluorescence was measured using Flow cytometry (Beckman Coulter Navios).

**Mitochondrial Calcium Measurement:** $5x10^5$ treated/control cells were stained with 5uM of Rhod-2 AM (ThermoFisher Scientific) for 20 minutes at 37 degrees. The stained cells were washed twice with phosphate-buffered saline at 500g for 5 minutes. The cell pellet was resuspended in PBS, and the fluorescence was measured using Flow cytometry (Beckman Coulter Navios).

**Untargeted Metabolomics:** 5 million cells were cultured and treated with DMSO, ATO, ART and the combination of ATO+ART. After 24 hours of treatment the cells were collected and quenched in a dry ice/ethanol bath followed by centrifugation for 10 minutes at 1000g, 4 degrees. The supernatant was removed completely, and the pellet was re-suspended in 200ul of (1:3:1) ice-cold Chloroform: Methanol: Water mixture to extract the metabolites and incubated for 1 hour in a rocker at 4 degrees. Then, the sample was spun at 13000g for 3 minutes at 4 degrees and the supernatant was collected and stored at -80 degrees. Liquid chromatography-mass spectrometry (LC/MS) for the extracted metabolites was performed using a pHILIC column in a Thermo Orbitrap at the Glasgow polyomics facility, University of Glasgow, UK.

**Supplementary Figure 1:**

a) The total number of colonies by the normal CD34-positive cells treated with ATO, ART, and ATO+ART at the end of 14 days cultured in the Methocult (H4434) (n=5; ATO =2uM; ART = 5uM)
b) Types of colonies formed by the normal CD34 positive post 14 days of ATO, ART and ATO+ART were characterized using FACS (StemMACS HSC-CFU Assay Cocktail). (n=5; ATO = 2 µM; ART = 5 µM).
c) Immunoblot of full-length caspase-3 (32kDa) and cleaved Caspase-3 (17kDa) in U937 cells treated with ATO and ART for 48 hr (n=3; ATO = 2 µM; ART = 5 µM).
d) Representative images of Wright-Giemsa-stained U937 cells treated with ATO, ART, and ATO+ART for 24h (ATO=2uM; ART=5uM).
e) Viability of U937 cells treated with ATO, ART and ATO+ART in combination with CB839 and etomoxir for 48h (n=6; ATO=2uM; ART=5uM and CB839= 10uM; ETO= 10uM).

(f-h) Relative expression levels of GLUT-1, CD36 and GLS in U937 cells treated with ATO, ART and ATO+ART. (GLUT-1 and CD36 - n=3; GLS n=6; ATO=2uM; ART=5uM).

(i-j) Reactive species levels of U937 cells treated with ATO, ART and ATO+ART for 6h and 24h (n=3; ATO=2uM; ART=5uM).

BFU-E: burst-forming unit-erythroid, CFU-G: colony-forming unit-granulocyte, CFU-M: CFU-macrophage, CFU-GM: CFU-granulocyte macrophage, CFU-GEMM: CFU-granulocyte erythrocyte macrophage megakaryocyte.
Data are presented as mean ± SEM. n.s., P > .05; ∗P < .05; ∗∗∗P < .001; ∗∗∗P < .0001, with a two-tailed unpaired t-test or one-way analysis of variance.

## Supplementary figure 1

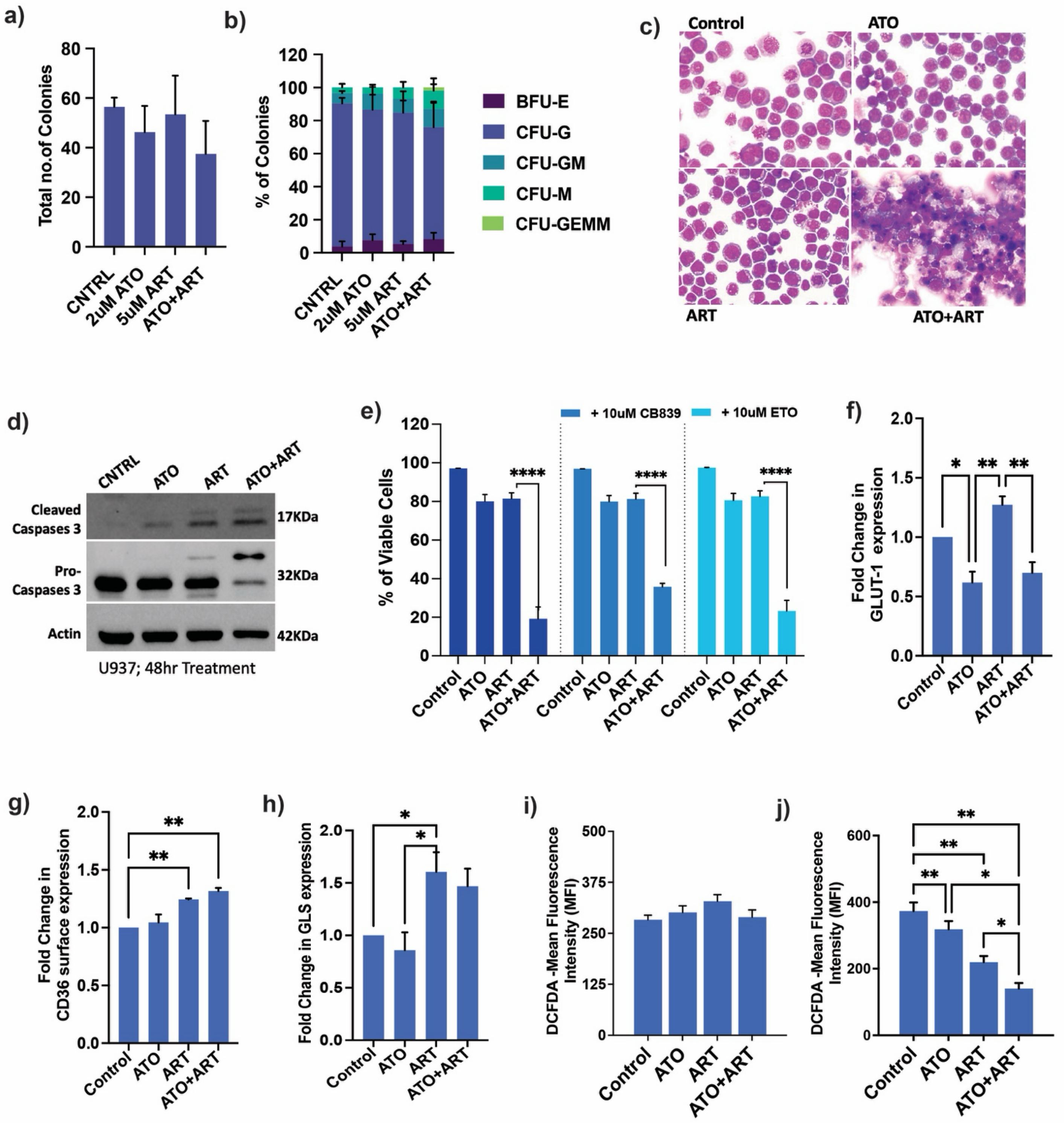

**Supplementary figure 2:**

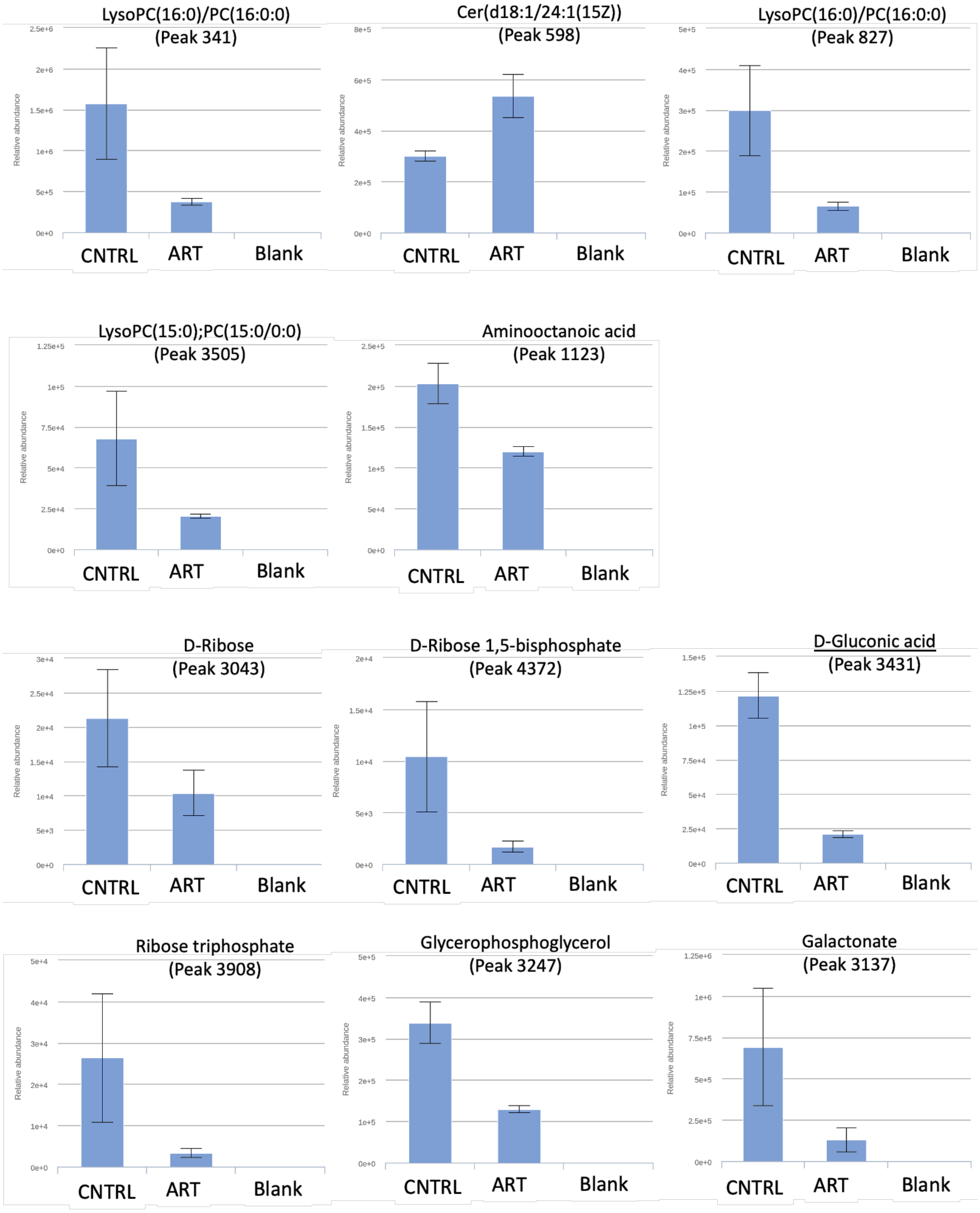


Untargeted metabolomics of U937 cells treated with ART for 24h: Relative abundance of metabolites relating to lysophospholipids, and sugars. (n=3; ART=5uM; 24h)

**Supplementary figure 3:**

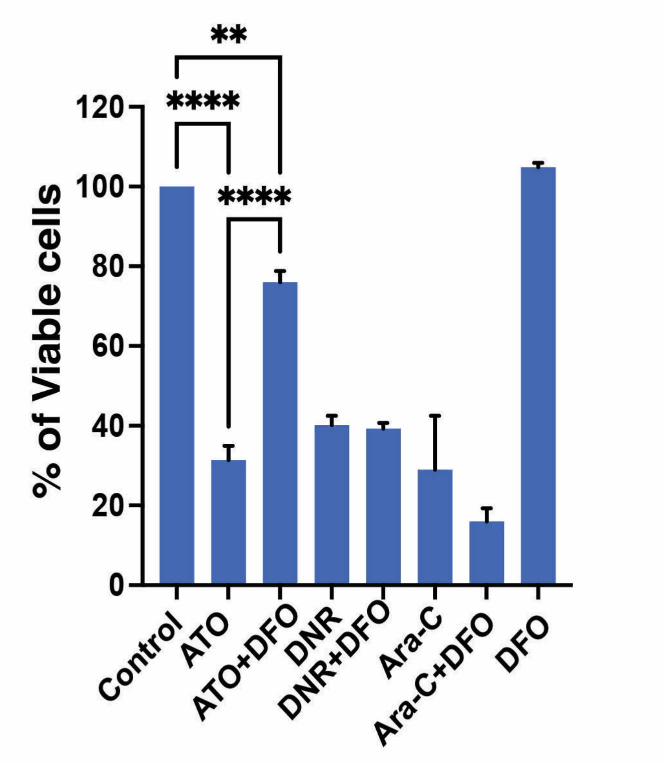


Viability of ATO sensitive NB4 cells treated with ATO, DNR, Ara-C in combination with DFO for 48h (n=3; ATO=2uM; DNR = 40ng; Ara-C =400ng; DFO=20uM).

Data are presented as mean ± SEM. n.s., P > .05; ∗P < .05; ∗∗∗P < .001; ∗∗∗P < .0001, with a two-tailed unpaired t-test or one-way analysis of variance.

**Supplementary Figure 4:**

a) b)

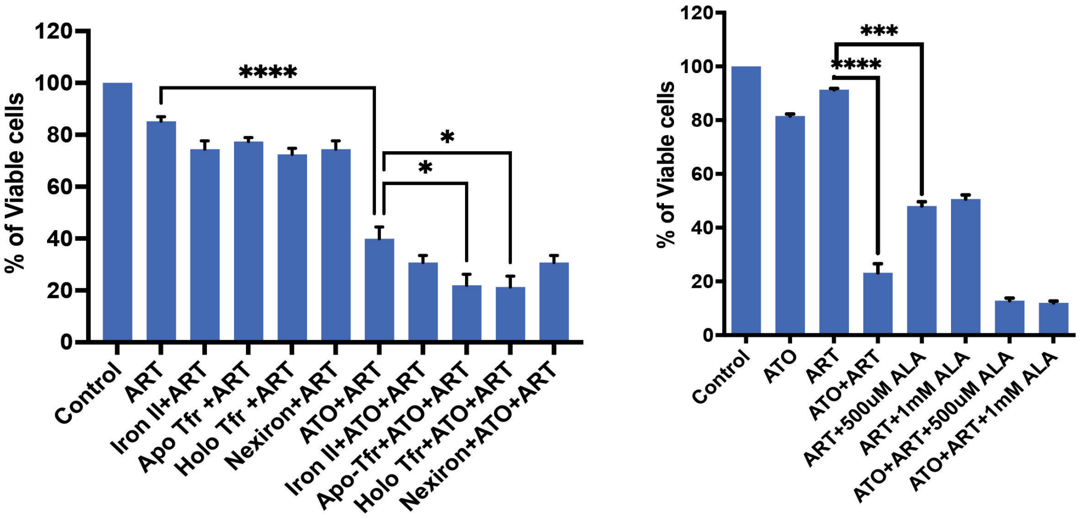


a) Viability of U937 cells treated with different forms of iron in combination with ART and ATO+ART for 48h. (n=3; Iron II = 10ug; Apo and Holo Tfr = 50ug; Nexiron = 40ug).

b) Viability of U937 cells treated with ALA in combination with ATO, ART, and ATO+ART for 48h. (n=4; ATO = 2uM; ART = 5uM and ALA = 500uM and 1mM)

Data are presented as mean ± SEM. n.s., P > .05; ∗P < .05; ∗∗∗P < .001; ∗∗∗P < .0001, with a two-tailed unpaired t-test or one-way analysis of variance.

**Supplementary Figure 5:**

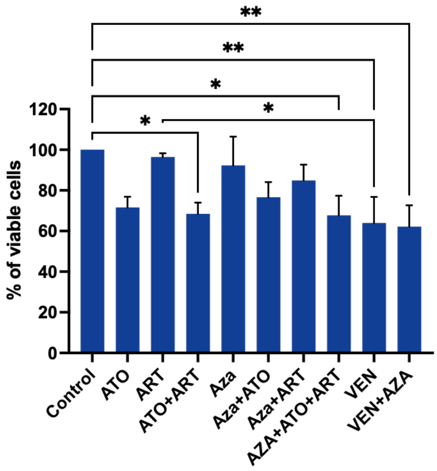


Viability of healthy peripheral blood mononuclear cells treated with ATO, ART, VEN and AZA for 48h. (n=5; ATO=2uM; ART =5uM; Aza =2.5ug; VEN = 500nM).

Data are presented as mean ± SEM. n.s., P > .05; ∗P < .05; ∗∗∗P < .001; ∗∗∗P < .0001, with a two-tailed unpaired t-test or one-way analysis of variance.

**Supplementary figure 6:**

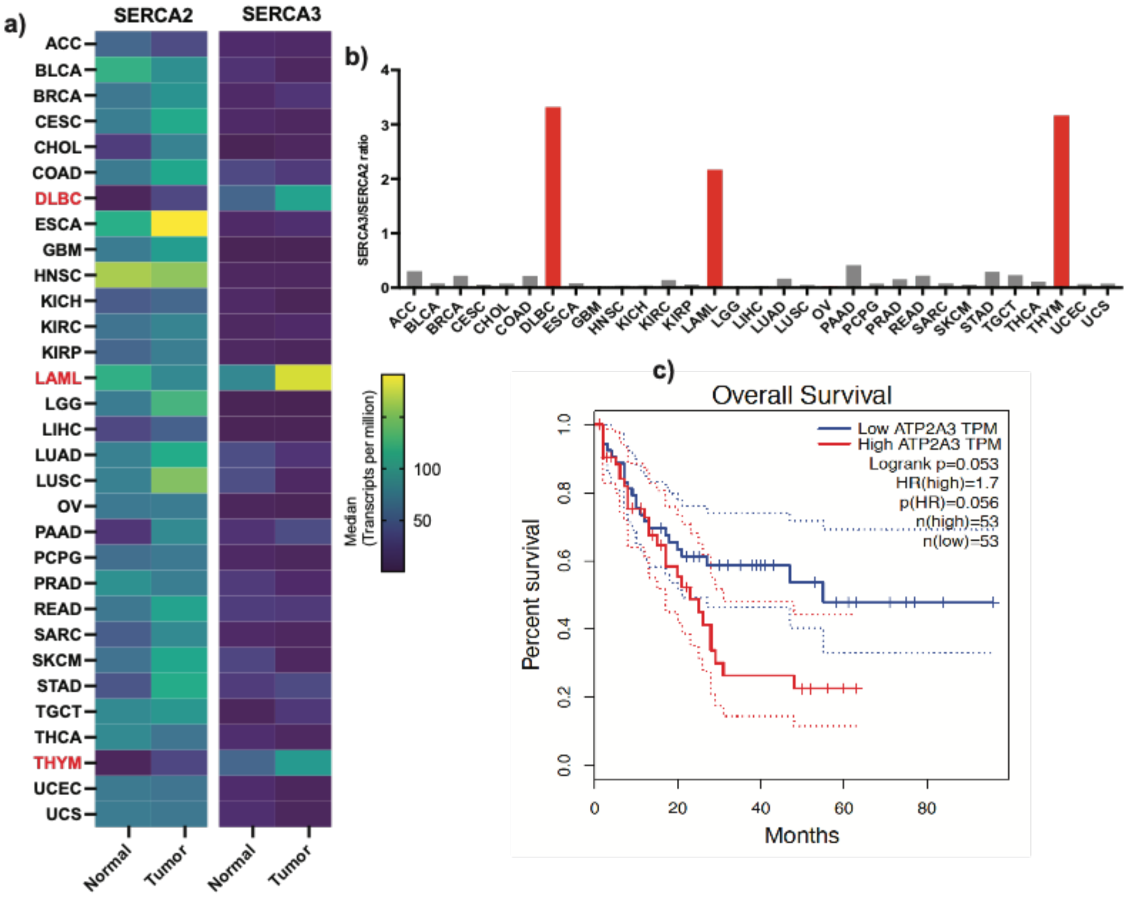


a) SERCA2 and SERCA3 expression levels of normal and cancer tissues from the Cancer Genome Atlas TCGA dataset.
b) SERCA3 to SERCA2 ratio for TCGA tumor types. Red bars highlight the hematological malignancies with a high SERCA3 to SERCA2 ratio. DLBC (Diffuse large B cell lymphoma), LAML (Acute myeloid leukemia), and THYM (Thymoma).
c) Kaplan Meier survival curves of TCGA AML cohort stratified based on SERCA3 expression.

**Supplemental Tables:**

**Table 1:** Relative protein abundances enriched in the biotinylated ART pull-down of U937 cells (related to figure 2b and c).

**Table 2:** Metabolites peak intensities of ART-treated U937 and control cells (related to figure 2d and Supplementary figure 2).
