# Supplementary material for: A novel strategy to target metabolic dependencies in acute myeloid leukemia
Source: Cell Death Dis. 2025 Nov 4;16(1):792. doi: 10.1038/s41419-025-08129-3 (PMC12586699; doi:10.1038/s41419-025-08129-3)

Corresponding to Figure 2e

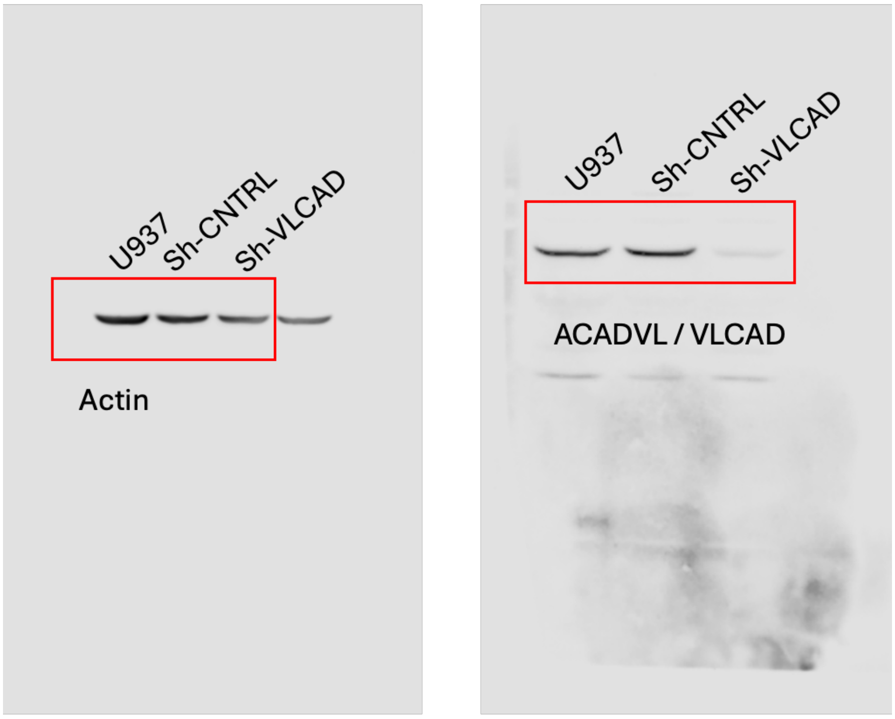

Corresponding to Figure 5c

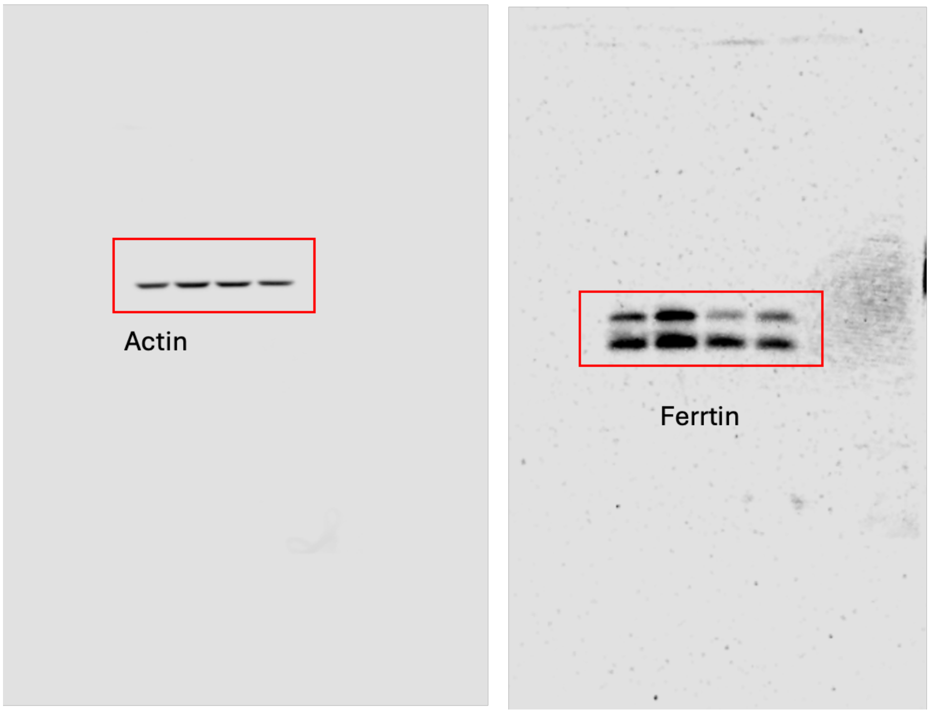

Corresponding to supplementary figure 1c

Caspase-3

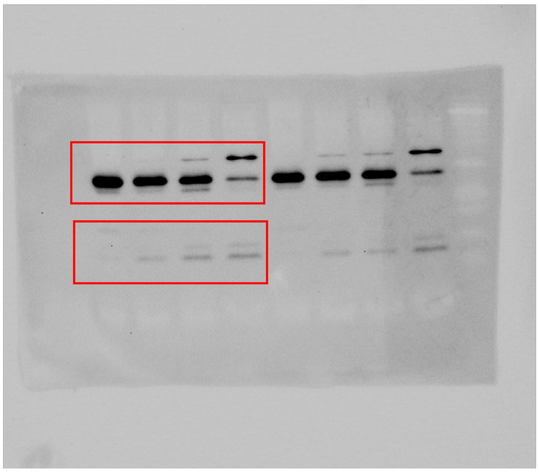

Actin

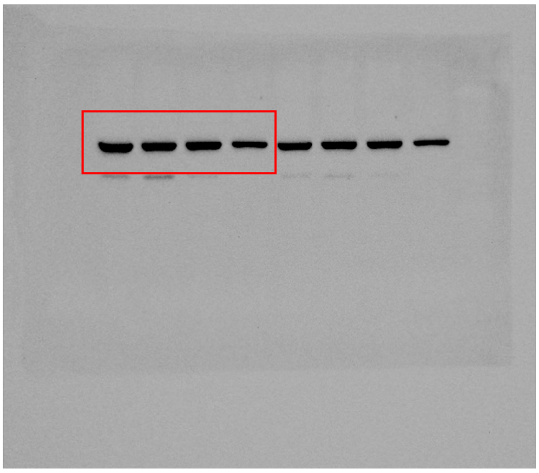

Supplement: Supplementary file 2 — Uncropped Westernblots [file 41419_2025_8129_MOESM2_ESM.pdf]
